# Supplementary material for: A high-resolution mRNA expression time course of embryonic development in zebrafish
Source: eLife. 2017 Nov 16;6:e30860. doi: 10.7554/eLife.30860 (PMC5690287; doi:10.7554/eLife.30860)
Supplement: Supplementary file 6. [file elife-30860-supp6.zip › biolayout-clusters-files/Cluster112-genes.html]

Cluster112


# Cluster112: Genes

| Ensembl ID | Gene Name | Chr | Start | End | Biotype |
| --- | --- | --- | --- | --- | --- |
| ENSDARG00000096601 | FAM13C | 12 | 7485180 | 7551113 | protein\_coding |
| ENSDARG00000074465 | VPS13C | 7 | 29362520 | 29452686 | protein\_coding |
| ENSDARG00000098161 | cntn4 | 6 | 45493129 | 45836252 | protein\_coding |
| ENSDARG00000040248 | crata | 5 | 32215950 | 32230889 | protein\_coding |
| ENSDARG00000099524 | gbe1b | 15 | 7408449 | 7622378 | protein\_coding |
| ENSDARG00000041068 | got2a | 18 | 21189655 | 21199702 | protein\_coding |
| ENSDARG00000016573 | mroh1 | 16 | 31301093 | 31387319 | protein\_coding |
| ENSDARG00000099730 | pkma | 18 | 1014890 | 1059194 | protein\_coding |
| ENSDARG00000059956 | ptk6b | 23 | 17460982 | 17477668 | protein\_coding |
| ENSDARG00000043848 | sod1 | 10 | 25732682 | 25737741 | protein\_coding |
| ENSDARG00000010010 | trim13 | 9 | 29832111 | 29844252 | protein\_coding |
